# Oral Ingestion of *Cannabis sativa*: Risks, Benefits, and Effects on Malaria-Infected Hosts

**DOI:** 10.1089/can.2018.0043

**Published:** 2018-11-26

**Authors:** Olugbenga Akinola, Elizabeth O. Ogbeche, Hidayah A. Olumoh-Abdul, Abdulmusawwir O. Alli-Oluwafuyi, Aboyeji L. Oyewole, Abdulbasit Amin, Wahab Imam AbdulMajeed, Olayemi Joseph Olajide, Abdurrazaq B. Nafiu, Anoka A. Njan, Olufunke E. Olorundare, Grace O. Gbotosho

**Affiliations:** ^1^ Department of Pharmacology and Therapeutics, Faculty of Basic Medical Sciences, University of Ilorin, Ilorin, Nigeria.; ^2^ Biomedical Research Group, University of Ilorin, Ilorin, Nigeria.; ^3^ Malaria Research Laboratories, Institute for Advanced Medical Research and Training, College of Medicine, University of Ibadan, Ibadan, Nigeria.; ^4^ Department of Pharmacology and Toxicology, Faculty of Pharmacy, University of Ilorin, Ilorin, Nigeria.; ^5^ Department of Physiology, University of Ilorin, Ilorin, Nigeria.; ^6^ Department of Anatomy, University of Ilorin, Ilorin, Nigeria.; ^7^ Department of Pharmacology and Therapeutics, University of Ibadan, Ibadan, Nigeria.; ^8^ Department of Pharmacology and Toxicology, Faculty of Pharmacy, University of Ibadan, Ibadan, Nigeria.

**Keywords:** *Cannabis therapeutics*, malaria, disease tolerance, *Plasmodium berghei*, asymptomatic reservoirs

## Abstract

**Background:** The emergence of a multidrug-resistant strain of *Plasmodium falciparum* (*Pf Pailin*) raises concern about malaria control strategies. Unfortunately, the role(s) of natural plants/remedies in curtailing malaria catastrophe remains uncertain. The claims of potential antimalarial activity of *Cannabis sativa in vivo* have not been well established nor the consequences defined. This study was, therefore, designed to evaluate the effects of whole cannabis consumption on malaria-infected host.

**Methods:** Thirty mice were inoculated with dose of 1×10^7^ chloroquine-resistant *Plasmodium berghei* ANKA-infected erythrocyte and divided into six treatment groups. Cannabis diet formulations were prepared based on weighted percentages of dried cannabis and standard mice diet and the study animals were fed *ad libitum*. Chemosuppression of parasitemia, survival rates, parasite clearance, and recrudescence time were evaluated. Histopathological studies were performed on the prefrontal cortex (PFC) and hippocampus of the animals after 14 days' consumption of cannabis diet formulation by naive mice.

**Results:** There was a significant difference (*p*<0.05) in the day-4 chemosuppression of parasitemia between the animals that were fed *C. sativa* and chloroquine relative to the untreated controls. There was also a significant difference in the survival rate (*p*<0.05) of animals fed *C. sativa* diet (40%, 20%, 10%, and 1%) in contrast to control animals on standard mice diet. A parasite clearance time of 2.18±0.4 was recorded in the chloroquine treatment group, whereas recrudescence in chloroquine group occurred on day 7. There were slight histomorphological changes in the PFC and cell densities of the dentate gyrus of the hippocampus of animals that were fed *C. sativa.*

**Conclusions:** *C. sativa* displayed mild antimalarial activity *in vivo.* There was evident reduction in symptomatic manifestation of malaria disease, though unrelated to levels of parasitemia. This disease tolerance status may be beneficial, but may also constitute a transmission burden through asymptomatic carriage of parasites by habitual cannabis users.

## Introduction

Continuing efforts at combating and eliminating malaria have culminated in the dramatic reduction of the malaria burden, with an estimated 6.2 million malaria deaths averted between 2001 and 2015.^1^ The successes are, however, being threatened by a number of factors, including parasite drug resistance.^2^ Increasing reports of reduced parasite susceptibility and drug failures in Southeast Asia due to parasite resistance to artemisinin-based combination therapies^3–9^ now threaten malaria control efforts. The clinical consequences of artemisinin resistance may be dire, and more devastating in Africa, where >90% of all malaria cases are recorded.^10,11^ More worrisome is the emergence of a multidrug-resistant strain of *Plasmodium falciparum* (*Pf Pailin*) from the Southeast Asia region now referred to as “Super Malaria.”^12^

The alarming rate of drug failure necessitates the development and deployment of novel and highly efficacious antimalarial drugs. It is thus not surprising that numerous ongoing researches focus on finding newer chemotherapeutic agents that can combat and counter drug-resistant parasites. The role/use of natural plant remedies in curtailing the ever-burgeoning malaria catastrophe remains uncertain, as only a small fraction of plants have been evaluated and developed for their medicinal potentials.^13^ However, many communities in malaria-affected regions still rely on natural plants and herbs, despite documented concerns about efficacy and adverse effects.^14^

One plant of continuous controversy is *Cannabis sativa*, known mainly for its psychoactive effects but possesses other activities.^15,16^ Quite a number of medicinal preparations derived from *C. sativa* have been employed for a variety of ailments, including malaria, pain, fever, and rheumatism.^17^ The constituents of cannabis have also been linked to modulation of the immune system,^18–22^ and these immune responses play major roles in the pathophysiology of inflammatory diseases.^23,24^ The uncontrolled and irrational use of *C. sativa* by individuals may create a challenge for the management of diseases.

Cannabis, also known as marijuana, pot, weed, gbanaa, and dagga in different regions, is consumed in different ways, including smoking, vaporizing, and as tea and edibles. Onset of effects is within minutes when smoked and about 30–60 min when cooked and/or eaten.^25^ The prevalence of cannabis use by persons remains unstable due to limited and unreliable data from most countries. However, estimated figures show a high rate (>10% of population) of use in 19 out of 163 countries where data were available.^26^

Isolated constituents of cannabis, 4-acetoxycannabichromene, 5-acetyl-4-hydroxycannabigerol, and -1′S hydroxycannabinol, have been reported to possess mild-to-moderate antimalarial activity *in vitro.*^27,28^ Information on the potential of *C. sativa* in suppressing malaria infections *in vivo* is sparse, although users in malaria endemic regions claim that cannabis protects against malaria.^29^ The exploration of the potential antimalarial properties of *C. sativa* consumption *in vivo* is necessary to establish the favorable and/or unwanted effects of *C. sativa* in malaria-infected hosts. This information will be vital, as cannabinoids the major constituent of *C. sativa* plant are known to bind tightly to their receptors found in the brain.^15,30^ It is important to identify potentially valuable quantities of cannabis that may be useful against malaria infections. In this study, we attempt to model daily consumption of *C. sativa* by oral ingestion *ad libitum*, and its potential effects on chloroquine-resistant *Plasmodium berghei*-infected mice.

## Materials and Methods

### Animals

Swiss albino mice of average weight of 24 g were utilized in all the experiments. The mice were used in accordance with the University of Ilorin “Guide for the care and use of laboratory animals.”

### Plants

Dried leaves, twigs, and seeds of the *C. sativa* plant were obtained from the National Drug Law Enforcement Agency (NDLEA), Kwara State Command, Nigeria. Identification, characterization, and validation of the plant were confirmed by evidence specialist at NDLEA, Kwara State Command.

### Preparation of cannabis-feed formulation

The cannabis-standard feed formulations were prepared based on weighted percentages of dried *C. sativa* (leaves, twigs, and seeds in the ratio 6:3:1, respectively) and standard mice feed. This was presented as food to study mice *ad libitum*. A percentage weighed portion of milled *C. sativa* was mixed with corresponding percentage quantities of milled standard mice feed for different groups corresponding to 40%, 20%, 10%, and 1% cannabis diet. The cannabis standard feed formulation was pelletized and labeled as “cannabis diet formulations” (*C. sativa* diet). The formulations were fed to study mice *ad libitum.*

### Determination of 50% lethal dose (LD_50_)

A modification of Lorke's toxicity test^31^ was employed using 12 naive mice separated into three groups (I–III) and fed with 20%, 2%, and 0.2% cannabis diet, respectively. The animals were closely monitored for signs of toxicity or lethality for 24 h, after which the cannabis diet formulation was withdrawn and monitoring continued for 14 days. In the second phase, five animals were fed with 50% cannabis diet formulation *ad libitum*. These animals were also monitored for toxicity as already stated. In both phases, the animals fasted overnight before being fed the cannabis diet formulation.

### Antimalarial test *in vivo*

A modified Peter's 4-day suppressive test^32^ was employed in assessing antimalarial activity. Thirty experimental animals were intravenously inoculated with a dose of 1×10^7^ chloroquine-resistant *P. berghei*-infected erythrocyte. The animals were separated into six treatment groups of five mice per group comprising a positive control group (chloroquine 10 mg/kg), a negative control group (water), and four groups based on percentages of cannabis in the diet formulation (40%, 20%, 10%, and 1%) in respective groups (I–IV). Chloroquine (10 mg/kg) treatment commenced 24 h postinoculation and administered once daily for 3 days. Animals in the cannabis groups fasted overnight and were allowed access to the cannabis diet formulation *ad libitum* 24 h post-inoculation and throughout the duration of study. Animals in the chloroquine treatment and negative control group were fed standard mice diet.

Parasiticidal activity was assessed daily from day 1 post-infection till day 14, and then on days 17 and 21. Inhibition of parasite growth in chloroquine-treated and cannabis diet groups were calculated relative to parasitemia in the negative control. Parasite clearance and recrudescence times were also evaluated. Survival rates in all experimental groups were monitored daily and the mean survival time was calculated.

### Assessment of hematological parameters/indices

Twenty naive mice were randomly divided into five groups of four animals each. The animals in groups I to IV were fed with cannabis diet formulation containing 40%, 20%, 10%, and 1% cannabis for 14 days. Animals in group V were fed with standard mice feed. The animals were euthanized, blood collected through cardiac puncture into potassium-ethylenediamine tetraacetic acid bottles, and immediately analyzed for hematological parameters.

### Histology of brain tissues

To avoid any potential effect of parasitemia or ambiguity of the action of *C. sativa* on brain tissues, uninfected naive mice were used for this experiment. Fifteen mice were randomly divided into five groups of three mice each. Animals in groups I to IV were fed with cannabis diet formulation containing 40%, 20%, 10% and 1% cannabis, respectively, for 14 days. Animals in group V were fed the standard mice diet. The animals were sacrificed by cervical dislocation, dissected frontally, and perfused with normal saline, to rid the tissues of blood. The whole brain was excised and rinsed in 30% sucrose solution, followed by fixation in 4% para formaldehyde in plain bottles placed on ice and later stored at 4°C. After 24 h, the prefrontal cortex (PFC) and hippocampus were excised and preserved in 30% sucrose solution at 4°C and later processed for histological staining with hematoxylin and eosin (H&E) and 0.1% cresyl fast violet (CFV).

### Statistics

Student *t*-test was used to analyze the differences in mean across all experiments. All experiments were conducted in triplicates and analyses were considered statistically significant at *p*<0.05.

## Results

### Lethal dose (LD_50_)

There were no signs of toxicity or lethality across all groups of mice fed with 50%, 20%, 2%, and 0.2% cannabis diet formulation *ad libitum* within 24 h. There was also no mortality recorded nor any observed sign of toxicity over a period of 2 weeks after the withdrawal of the cannabis diet even at the highest dose of 50% *C. sativa* in diet formulation.

### Assessment of intrinsic antimalarial activity

**FIG. 1.**
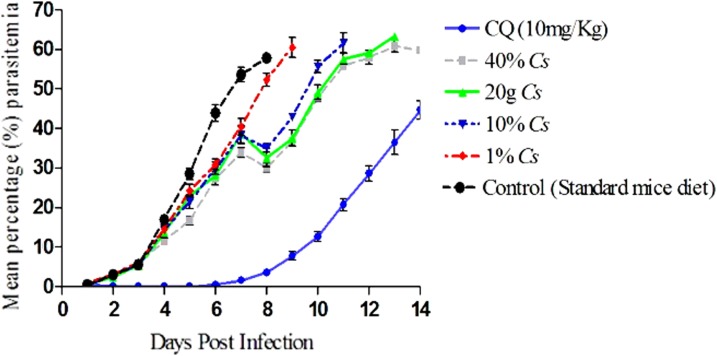
Comparative response of treatment with chloroquine, oral ingestion of *Cannabis sativa*, and controls (standard mice diet) against chloroquine-resistant *Plasmodium berghei* ANKA strain parasites in mice.

The susceptibility profile of the parasites to cannabis, chloroquine treatment, and in the untreated negative controls is described in Figure 1. Parasitemia in the untreated control animals ranged from 17.01% on day 4 post-infection to a peak of 57.85% on day 8 when the last animal in the group died. There was no significant difference (*p*>0.05) in the day-4 suppression of parasitemia between the group of animals that were fed 1%, 10%, and 20% *C. sativa* diet formulation and the untreated controls (Fig. 1). However, there was a significant increase in day-4 parasite suppression in the group of animals that were fed with 40% *C. sativa* formulation (*p*=0.001) relative to the control. There was a quantity-dependent effect in the day-4 mean percentage chemosuppression across the groups with 31.68%, 18.98%, 15.22%, and 14.16% suppression of parasitemia in animals from groups I to IV, respectively. There was complete suppression of parasites on day 4 in the chloroquine-treated group. Parasite clearance was recorded in the chloroquine treatment group with a mean parasite clearance time of 2.18±0.4 days and recrudescence time of 6.80±0.6 days. A reduction in parasitemia though not significant was observed on day 8 postinfection in the cannabis treatment groups of 40%, 20%, 10%, and 1% cannabis diet.

### Survival rate of study animals

The day 7 survival rates of animals fed on the cannabis diet formulation were consistent with the survival rates of chloroquine-treated animals (Table 1), despite the presence of significantly higher parasitemia in animals fed on cannabis diet formulation. There were significant differences in the day 14 survival rates between chloroquine treatment group and *C. sativa* treatment groups of 20%, 10%, and 1% *C. sativa* diet. However, day 14 survival rates of animals fed with 40% *C. sativa* diet formulation were comparable with those with chloroquine-treated animals. The day 14 survival rate indicated a quantity-dependent effect of *C. sativa* consumption. There was a comparable mean survival time between the chloroquine treatment and the group of animals that consumed 40% cannabis diet. There was also a significant difference (*p*<0.05) in mean survival time between all treatment groups and the untreated control (Table 1).

**Table 1.** **Mean Survival Time and Percentage Survival of Malarial-Infected Animals Fed with *Cannabis sativa***

	Percentage survival (%)		Mean survival time (days)
Treatment group	Day 7	Day 14	
Chloroquine (10 mg/kg)	100	100	17.2±1.8
40% *C. sativa*	100	60	15.2±2.3
20% *C. sativa*	100	20	12.8±1.4
10% *C. sativa*	100	0	11.2±1.8
1% *C. sativa*	100	0	9.4±1.3
Control (standard mice diet)	60	0	7.6±1.1

### Hematological indices

There was no statistical significance (*p*<0.05) in the hematological indices across all groups fed with 40%, 20%, 10%, and 1% cannabis diet formulations compared with the untreated controls fed standard mice diet. There was a remarkable decrease in platelet aggregation in the group of animals that were fed 40% *C. sativa* diet compared with other groups and controls, although this decrease was not significant. Table 2 gives the mean values of hematological parameters assayed in the different groups.

**Table 2.** **Hematological Parameters Assessed in Animals Fed with Varying Quantities of *Cannabis sativa* Diet Formulation *ad libitum* for 14 Days and Controls Fed Standard Mice Diet**

	Mean±SEM				
Parameter	40% *C. sativa*	20% *C. sativa*	10% *C. sativa*	1% *C. sativa*	Controls
WBC	3.12e^3^±1.1e^3^	3.0e^3^±1.3e^3^	6.7e^3^±1.6e^3^	3.4e^3^±7.5e^3^	3.5e^3^±4.2e^3^
RBC	7.06e^6^±7.7e^5^	5.66e^6^±1.1e^5^	7.47e^6^±1.6e^5^	5.54e^6^±7.3e^5^	7.12e^6^±9.2e^5^
PLT	6.23e^4^±8.9e^4^	5.72e^5^±1.3e^5^	6.43e^5^±2.8e^5^	6.39e^5^±1.6e^5^	7.16e^5^±2.5e^5^
LYM	2.8e^3^±9.5e^2^	4.45e^3^±1.8e^3^	5.75e^3^±1.3e^3^	2.78e^3^±5.4e^2^	3.1e^3^±3.2e^2^
HCT	30.63±1.16	28.13±6.27	35.58±1.45	34.60±2.90	2.90±4.25
MCV	48.50±1.12	47.90±0.95	47.57±0.91	47.43±1.01	47.93±0.72
MCH	13.20±0.12	12.97±0.56	13.40±0.27	12.97±0.32	12.87±0.38
MCHC	27.20±0.69	26.93±0.67	28.20±0.47	27.33±0.69	26.83±0.44
RDW	30.03±1.66	28.00±0.47	29.43±0.09	28.47±0.68	30.07±0.37
PDW	7.83±0.09	8.73±0.23	7.13±0.20	8.53±0.52	8.02±0.26
MPV	6.40±0.06	6.57±0.09	5.90±0.10	6.60±0.25	4.85±0.18
PLCR	5.30±0.12	5.37±0.52	3.67±0.50	5.33±0.89	4.62±0.55
HGB	7.30±0.95	7.40±1.63	10.03±0.38	9.23±1.27	9.13±1.30

HCT, total hematocrit; HGB, hemoglobin levels; LYM, lymphocyte count; MCH, mean corpuscular hemoglobin; MCHC, mean corpuscular hemoglobin concentration; MCV, mean corpuscular volume; MPV, mean platelet volume; PDW, platelet distribution width; PLCR, platelet large cell ratio; PLT, platelet count; RBC, red blood cell count; RDW, red cell distribution width; SEM, standard error of mean; WBC, white blood cell count.

### Histology of the PFC

**FIG. 2.**
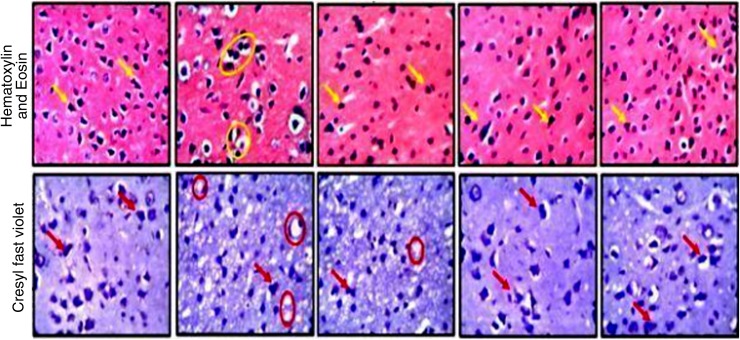
Representative photomicrographs of the prefrontal cortex of animals fed cannabis diet and untreated controls. A high power magnification showing the general histomorphology (H&E) and Nissl body characteristics (CFV) of animals fed 40%, 20%, 10%, and 1% for 14 days. The pyknotic cells are identified by the *yellow arrows*, while the chromatolytic neurons are captured in *red circles*. The chromatolytic neurons were also captured in *yellow circles* but under H&E staining, while the *red arrows* point to the pyknotic cell under the CFV staining. CFV, cresyl fast violet; H&E, hematoxylin and eosin.

Histological features of the animals in the control group did not show any altered morphology in panoramic presentation of the PFC layers. The cellular density within the control group appeared normal across all the layers with appreciable cellular projections. Animals fed 40% and 20% cannabis diet formulation showed slight changes in a quantity-dependent manner. These changes can be described by fragmented cells with observable pyknotic cells (yellow arrows), which is also associated with pronounced reduction of neuronal density in the 40% cannabis diet group. Animals in these groups (40% and 20% *C. sativa*) also presented with chromatolytic neurons (red circle) with reduced/irregular Nissl body distribution that suggests proteolysis. Groups of animals fed with 10% and 1% *C. sativa* showed characteristically normal cellular density and typical histoarchitectural assortment. Figure 2 depicts the photomicrographs showing the general cytoarchitecture of the PFC of animals fed 40%, 20%, 10%, and 1% *C. sativa* diet for 14 days with H&E staining and CFV stains.

### Histology of the hippocampus

**FIG. 3.**
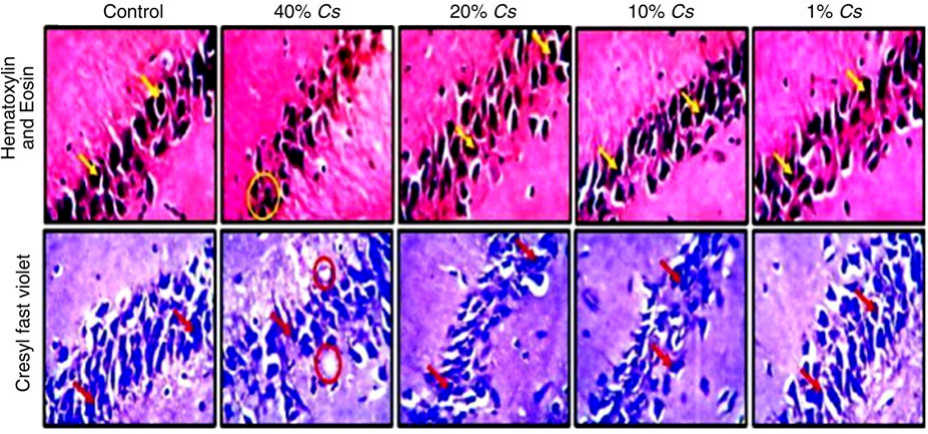
Representative photomicrographs of CA region of the hippocampus of animals fed cannabis diet and untreated controls. A high power magnification showing the general the histomorphology (H&E) and Nissl body characteristics (CFV) stains of animals fed 40%, 20%, 10%, and 1% *Cannabis sativa* for 14 days. The large pyramidal cells are identified by *yellow arrows*, while the pyknotic cells can be spotted in *yellow circles*. The *red arrows* point at the large pyramidal cell, while the *red circles* show the pyknotic cells, both under CFV staining. CA, cornu ammonus.

The control group did not show any alteration in morphological presentation of the hippocampal layers across the various exposures and magnification (×400). Groups of animals fed with 20%, 10%, and 1% *C. sativa* showed normal architecture, characterized by large pyramidal cells (yellow arrows) with appreciable axonal projections across the intact neuropil. The histomorphology of the hippocampus in animals fed 40% *C. sativa* diet displayed moderate-to-mild chromatolytic changes characterized by clusters of pyknotic cells (yellow circles) and reduced neuronal density relative to the control group. Figure 3 shows the pictorial representation of the hippocampus stained with H&E and Nissl profile demonstration by CFV stain (×400) across hippocampal sections of the brain in the group of animals labeled control (standard diet), 40%, 20%, 10%, and 1% *C. sativa* diet formulation for 14 days.

## Discussion

*C. sativa* is considered the most widely consumed illicit substance,^26^ largely so because of its usefulness in ameliorating a wide variety of ailments.^17^ The findings of this study outline the beneficial/risk interactions of whole *C. sativa* ingested orally by malaria-infected hosts. In this study, confiscated dried *C. sativa* was milled and mixed with standard mice feed and presented as food to mice *ad libitum*, to model the effects of oral cannabis consumption in habitual users. Dried leaves, twigs, and seeds were blended to mimic the preparations of cannabis in many places in Nigeria, where users often claim its protective potentials against infectious diseases such as malaria, alongside its psychoactive effects. In our survey of herbs in the treatment of malaria, cannabis is neither considered nor listed as part of ethnobotanical treatment of malaria in any part of Nigeria (unpublished).

Oral ingestion of *C. sativa* is the second most common method of *C. sativa* consumption, probably due to lack of toxic manifestations. This was demonstrated in this study with animals consuming blends of whole *C. sativa* up to 50% in the feed formulation for 24 h without any observable toxic concerns. This is corroborated by previous findings^33^ that assessed the tolerability of oral acute dose extracts of *C. sativa,* tetrahydrocannabinoids and cannabinoids, in humans. Also, cannabis is largely considered nontoxic, as no deaths have ever been reported due to cannabis overdose or unintended overconsumption, though it may possess risk for individuals with long-term use.^25^

*C. sativa* demonstrated poor *in vivo* chemosuppressive activity across all groups with mild-to-moderate antiparasitic activity in study animals. The phytocannabinoid metabolites of *C. sativa* may have contributed to the moderate antimalarial activity, as these compounds have been shown to possess antiplasmodial action *in vitro.*^27,28^ However, there might be other constituents of *C. sativa* that may have contributed to^34^ or reduced its activities by metabolic influences *in vivo,* as *C. sativa* is reported to mediate negative or positive characteristics on different pathological states.^30,35,36^ *C. sativa* exhibited a delayed suppressive effect on parasites in a quantity-dependent manner, as shown in our data from day 4 onward. The possibilities of bioaccumulated *C. sativa* constituents and/or its metabolites contributing to an improved parasite suppression were ruled out, as the animals were allowed continuous access to *C. sativa*, but there was no significant reduction in parasitemia until death.

*C. sativa* ingestion significantly increased the survival of infected animals in a quantity-dependent manner. Interestingly, the animals were not devoid of the high parasitic burden associated with malaria pathogenesis in mice. Although the antiparasitic action of *C. sativa* may not be comparable with that of chloroquine, the similarities in mean survival time in the *C. sativa* group fed 40% *C. sativa* may suggest the potential of cannabis in reducing pathogenicity of *P. berghei*. The disease tolerance status may not be unrelated to the immunomodulatory (pro- and anti-inflammatory) properties of cannabis,^19,22,37,38^ as have been shown in a study on cerebral malaria.^39^ The various properties from many different constituents of cannabis may have effected a trade-off of natural immune responses^40^ in the animals during malaria infections. This assertion is supported by previous reports that life-threatening manifestations of malaria are often as a result of dysregulation of host immune mechanisms.^41,42^ Irrespective of the potentials of ingested *C. sativa* to reduce the disease burden, infected animals eventually succumbed to malaria-related death. This shows that *C. sativa* is not curative against malaria infections, but may possess activity to enhance disease tolerance. Although death may also have been aided by the pathophysiological effects of *C. sativa* at quantities consumed by the study animals, the mechanism(s) involved remain(s) to be addressed.

Malaria parasites are known to preferentially invade and depend on the erythrocytes of the host for survival and proliferation. The influence of cannabis consumption on the blood cell integrity is necessary to assess the parasite infectivity profile in hosts. There were, however, no hematological changes in naive mice fed with cannabis to suggest any mechanism modulating invasion of red cells that may affect parasitemia.

The potential of cannabidiol to prevent behavioral changes in mice infected with *P. berghei ANKA* has been reported.^39^ In this study, the histomorphology of the PFC and hippocampus of naive animals showed evidence of morphological changes in a quantity-dependent manner as previously suggested.^43^ These slight changes may be manifestations of the deleterious effects of *C. sativa* consumptions on the brain tissues,^30^ although it remains inconclusive as other adjuncts on the *C. sativa* plant may also be responsible. Although not assessed in this study, manifestations of PFC and the Hippocampal degeneration, such as, impaired motor function, short-term memory, paranoia, and psychosis may be apparent as previously reported.^15^ However, these degenerative effects may not play a role in the pathogenesis of cerebral malaria in cannabis users as the pathways and processes differ.

Observations from this study show the potential benefit of cannabis to mediate a disease tolerance effect during malarial infection, as demonstrated by the reduced negative effect of parasitemia on the animals. However, the host may become an active reservoir of the malaria parasite, capable of onward transmission of parasites within the population. This may pose a setback to malaria elimination, as it may contribute to potential resistance and cross-resistance from suboptimal concentrations of the mildly active antiplasmodial properties of *C. sativa*. The host may also become an “asymptomatic pool,” harboring parasites that may become more virulent upon transmission to noncannabis users, as pathogens from asymptomatic carrier have been reported to be more virulent on symptomatic hosts.^44,45^

Although our study assessed the use of dried whole cannabis plant to model a common form of ingestion, the use of cannabis inflorescence may have produced a better antimalarial activity. Also, the ingestion route may have occluded the release of some other potential antimalarial constituents of cannabis, such as in its essential oils that contain high levels of terpinoids. Terpineol isolated from the oil of Tetradenia riparia plant has previously been reported to possess antimalarial activities *in vitro.*^46^ The variability in the chemical constituents of cannabis between cultivars may also produce slightly different results. Although there are several reports on the therapeutic use of cannabis constituents that can be validated, however, the consumption of whole cannabis is more prevalent globally.

## Conclusion

The potential of cannabis to reduce pathogenic manifestation of malaria disease in animals was established. However, there is the possibility of potential deleterious effects against malaria control efforts, thus, the continuous ingestion of cannabis blends may constitute more risk than benefits. Further research is needed to identify and isolate the different constituents of cannabis that may have mediated the benefits described in this study. Such findings may offer potential arrays into the modulation of vaccine activity and therapeutics that may limit the pathogenesis of severe and cerebral malaria incidences. With the new guidelines from the American Herbal Pharmacopeia on identification and standardization of cannabis for research, our understanding of this controversial plant will become clearer.

## Declaration on Data Set

We declare that the data supporting the conclusions in this work are submitted with this article. Primary/raw data set will be made available on request.

## Abbreviations Used

CFV: cresyl fast violet
H&E: hematoxylin and eosin
NDLEA: National Drug Law Enforcement Agency
PFC: prefrontal cortex
